# Recombinant bromelain production in *Escherichia coli*: process optimization in shake flask culture by response surface methodology

**DOI:** 10.1186/2191-0855-2-12

**Published:** 2012-02-15

**Authors:** Bala Muntari, Azura Amid, Maizirwan Mel, Mohammed S Jami, Hamzah M Salleh

**Affiliations:** 1 Bioprocess and Molecular Engineering Research Unit, Department of Biotechnology Engineering, Faculty of Engineering, International Islamic University Malaysia, P.O. Box 10, 50728, Kuala Lumpur, Malaysia; 2 Department of Biochemistry, Faculty of Science, Bayero University, Kano. P.M.B. 3011, Kano, Nigeria

**Keywords:** bromelain, *Escherichia coli *BL21-AI, face centered central composite design, induction.

## Abstract

Bromelain, a cysteine protease with various therapeutic and industrial applications, was expressed in *Escherichia coli*, BL21-AI clone, under different cultivation conditions (post-induction temperature, L-arabinose concentration and post-induction period). The optimized conditions by response surface methodology using face centered central composite design were 0.2% (w/v) L-arabinose, 8 hr and 25°C. The analysis of variance coupled with larger value of R^2 ^(0.989) showed that the quadratic model used for the prediction was highly significant (*p *< 0.05). Under the optimized conditions, the model produced bromelain activity of 9.2 U/mg while validation experiments gave bromelain activity of 9.6 ± 0.02 U/mg at 0.15% (w/v) L-arabinose, 8 hr and 27°C. This study had innovatively developed cultivation conditions for better production of recombinant bromelain in shake flask culture.

## Introduction

The use of highly purified proteins for therapeutic purposes has been in existence for many decades (Paul, 2004). Enzymes, mostly proteases, constitute the largest portion of these purified proteins for industrial and therapeutic applications. Proteases are enzymes that catalyze the hydrolysis of peptide linkages in proteins. They have wide applications in food, pharmaceutical and detergent industries. In fact, these enzymes constitute about 60% of all commercial enzymes in the world (Lucia and Tomas, 2010). Recently, microbial enzymes have been substituting those from other sources and might now account for almost 90% of the total market (Illanes, 2008). This is due to the fact that microbial cells are excellent systems for enzyme production. Thus, there is a great stimulation for extensive research works on recombinant proteins (Illanes, 2008).

Bromelain is a general name given to the family of sulfhydryl proteolytic enzymes (cysteine proteases) obtained from the pineapple plant, *Ananas comosus*. Depending on the source, it is usually classified as either fruit bromelain or stem bromelain (Kelly 1996). The sulfhydryl proteolytic fraction is the primary component of bromelain. The pineapple enzyme also contains several protease inhibitors, a peroxidase, acid phosphatase, and organically bound calcium (Kelly, 1996).

A member of papain family, stem bromelain (E.C.3.4.22.32) contains 212 amino acid residues including seven cysteines, one of which is involved in catalysis (Bitange *et al*., 2008). Pure stem bromelain is stable when stored at -20°C and has an optimum pH range of 6-8.5 for most of its substrates (casein, gelatin, synthetic peptides, etc.). The optimum temperature range for the enzyme is 50-60°C. It is mostly activated by cysteine while hydrogen sulfide and sodium cyanide are less effective (Bencucci *et al*. 2011). However, heavy metals such as mercury and silver, and L-*trans*-epoxysuccinyl-leucylamido (4-guanidino) butane [also known as E-64] deactivate the enzyme (Maurer, 2001). In contrast, fruit bromelain (E.C. 3.4.22.33) is genetically distinct from stem bromelain. It has higher proteolytic activity and broader specificity for substrates compared to stem bromelain (Maurer, 2001). Bromelain has been widely used in meat tenderization and as a dietary supplement (Ravindra *et al*. 2008), as well as food processing and baking industry (Lyons, 1982). Bromelain also has greater therapeutic applications. It was firstly introduced as a therapeutic compound in 1957 (Gregory and Kelly, 1996). Clinical applications of bromelain includes modulation of tumor growth, third degree burns, improvement of antibiotic action, etc. (Maurer, 2001).

Response surface methodology (RSM) has been greatly used for the optimization and studying the interactions among various bioprocess parameters using a minimum number of experiments. It is a unit of statistical tools for designing experiments, constructing models, assessing the effects of factors, and exploring optimum conditions of factors under study for desirable responses (de-Coninck *et al*. 2000). The technique has been widely utilized in many areas of biotechnological processes such as in the production of enzymes and antibiotics (de-Coninck *et al*. 2000).

*Escherichia coli *has been continuously utilized for the high-level production of recombinant proteins (Benucci, 2011). This is because of its availability and fully understood genetics. In addition, *E. coli *has the capacity to grow rapidly at high cell concentrations using cheap media (Manderson *et al*. 2006). Recombinant proteins expression in *E. coli *often leads to the formation of insoluble or nonfunctional proteins (Sørensen and Mortensen, 2005). The recovery of soluble protein from the inclusion bodies often yields less active enzyme and can significantly raise the cost of bioseparation (Lilie *et al*. 1998). Consequently, it is vital to express the protein in a biologically active form. Many factors affecting culture growth rates are being manipulated in order to reduce inclusion bodies formation. These include lowering of culture temperature (Hoffmann and Rinas, 2001), using early induction time of expression (Lim *et al*. 2000), nutrient and oxygen restriction (Ryan *et al*. 1989), increasing post-induction time, and regulating the inducer concentration (Manderson *et al*. 2006).

Considering the significance of assessing the effects of process variables on the recombinant proteins expression, the current research work was geared towards the evaluations of the effects of cultivation conditions (post-induction temperature, inducer concentration and post-induction period) on the production of soluble and active recombinant pineapple stem bromelain in *E. coli*.

## Materials and methods

### Chemicals

L-Cysteine, L-arabinose and casein were purchased from Sigma Chemicals Company (USA). Luria Bertani (LB) growth media used was a product of Merck, Germany. All other chemicals used were of analytical grade.

### Strain and plasmid

The *Escherichia coli *BL21-AI strain (Invitrogen, USA) harboring pineapple stem bromelain gene used in this study was described in our earlier study (Amid *et al*. 2011). Briefly, the gene encoding pineapple stem bromelain was initially cloned into pENTR/TEV/D-TOPO before being sub-cloned into the expression vector pDEST17 (Invitrogen, USA). The expression vector containing recombinant bromelain gene was then transformed in *E. coli *BL21-AI competent cells.

### Screening of cultivation conditions

The screening experiments were conducted in 250 mL shake flasks. Transformant cells were grown overnight in 5 mL LB media containing 100 μg/mL ampicillin until the OD_600 nm _reached 0.6-1.0 (Amid *et al*. 2011). One milliliter of the culture was then diluted 50 fold in a fresh LB media and then cultivated (at 37°C, 250 rpm) up to OD_600 nm _0.4-0.8, cell densities. In order to study the effects of post-induction temperature, the cultivation temperature was adjusted to 20-37°C (Table 1) and then L-arabinose was added to a final concentration of 0.2% (w/v) to each flask. For the inducer trial, the growth culture was adjusted to 37°C and then induced with 0.1-0.3% (w/v) L-arabinose. Post-induction period for cell harvest was studied at a range of 2-10 hr as presented in Table 1. All the experiments were conducted in triplicates.

**Table 1:** Induction parameters screened for bromelain production in BL21-AI

**Induction condition**^**1**^	**Range**^**2**^
Cell concentration (induction time)	OD_600 nm _of 0.4 - 0.8
L-arabinose concentration	0.1 - 0.3% (w/v)
Post-induction period (harvest time)	2 - 10 hr
Temperature	20 - 37°C

^1^All the tests were carried out at 37°C, OD_600 nm _of 0.4, 0.2% (w/v) L-arabinose and 4 hr post- induction period (besides the specific test conditions).

^2^Cultivation conditions and their ranges were selected based on literature reports on recombinant protein expression.

### Response surface methodology (RSM)

RSM was employed to optimize the screened induction conditions that enhanced the recombinant bromelain production. Face centered central composite design (FCCCD) developed by the Design Expert software (Version 6.0.8, Stat-Ease Inc., Minneapolis, USA), was used to optimize the three significant cultivation conditions: post-induction temperature, inducer concentration and post-induction period. A set of 20 experimental runs with six replicated center points were generated. Three different levels, low (-1), medium (0) and high (+1) were used to study the independent variables. The experimental design used for the study is presented in Table 2. All the experiments were carried out in triplicates and the average of bromelain activity was considered as the response (Y). The following second-order polynomial equation explains the relationship between dependent and independent variables:

**Table 2:** Experimental design used in RSM studies by using three independent variables for bromelain production

Run	Temperature,°C	**Inducer concentration**, % w/v	Post induction period, hr	Bromelain production (U/mg)	
				
				Experimental	Predicted
1	25 (0)	0.20 (0)	8 (0)	8.90 ± 0.08	9.10
2	20 (-1)	0.10 (-1)	6 (-1)	5.00 ± 0.03	4.90
3	30 (+1)	0.30 (+1)	10 (+1)	4.80 ± 0.02	4.90
4	20 (-1)	0.30 (+1)	6 (-1)	6.90 ± 0.05	6.90
5	25 (0)	0.10 (-1)	8 (0)	8.70 ± 0.08	8.70
6	25 (0)	0.20 (0)	8 (0)	9.20 ± 0.09	9.10
7	25 (0)	0.20 (0)	10 (+1)	8.50 ± 0.07	8.60
8	30 (+1)	0.10 (-1)	10 (+1)	8.50 ± 0.06	8.50
9	25 (0)	0.20 (0)	8 (0)	9.20 ± 0.09	9.10
10	30 (+1)	0.30 (+1)	6 (-1)	7.20 ± 0.05	7.20
11	25 (0)	0.20 (0)	8 (0)	8.90 ± 0.08	9.10
12	25 (0)	0.20 (0)	6 (-1)	8.60 ± 0.06	8.50
13	20 (-1)	0.30 (+1)	10 (+1)	7.30 ± 0.04	7.10
14	25 (0)	0.20 (0)	8 (0)	8.90 ± 0.07	9.10
15	20 (-1)	0.10 (-1)	10 (+1)	7.40 ± 0.05	7.40
16	25 (0)	0.30 (+1)	8 (0)	7.90 ± 0.06	7.90
17	25 (0)	0.20 (0)	8 (0)	9.20 ± 0.08	9.10
18	30 (+1)	0.10 (-1)	6 (-1)	8.40 ± 0.05	8.50
19	20 (-1)	0.02 (0)	8 (0)	7.60 ± 0.05	7.90
20	30 (+1)	0.20 (0)	8 (0)	8.80 ± 0.07	8.60

(1) $$\text{Y}=\beta_{0}+\beta_{1}{\text{X}}_{1}+\beta_{2}{\text{X}}_{2}+\beta_{3}{\text{X}}_{3}+\beta_{12}{\text{X}}_{\text{I}}{\text{X}}_{2}+\beta_{13}{\text{X}}_{\text{I}}{\text{X}}_{3}+\beta_{23}{\text{X}}_{2}{\text{X}}_{3}+\beta_{11}{\text{X}}_{1}^{2}+\beta_{22}{\text{X}}_{2}^{2}+\beta_{33}{\text{X}}_{3}^{2}$$

where Y is the dependent variable (bromelain production); X_1_, X_2 _and X_3 _are independent variables (temperature, inducer concentration and time, respectively); β_0 _is an intercept term; β_1_, β_2 _and β_3 _are linear coefficients; β_12_, β_13 _and β_23 _are the interaction coefficients; and β_11_, β_22 _and β_33 _are the quadratic coefficients. The evaluation of the analysis of variance (ANOVA) was determined by conducting the statistical analysis of the model. In order to depict the relationship between the responses and the experimental levels of each of the variables under study, the fitted polynomial equation was expressed in the form of contour and response surface plots.

### Validation of the experimental model

The statistical model was validated with respect to the entire three variables within the design space. A set of six experimental combinations, selected as predicted by the point prediction feature of the Design Expert software, were utilized to study the maximum bromelain production under defined conditions. All the experiments were carried out in triplicates and the results were then compared with the predicted values.

### Recombinant bromelain production with face centered central composite design (FCCCD)

The effects of varying post-induction temperature, L-arabinose inducer concentration and post-induction period on bromelain production were evaluated in shake flask experiments. As described earlier, all cultures were grown at 37°C and 250 rpm agitation until OD_600 nm _reaches 0.6. This was followed by adding varying L-arabinose concentrations (0.1-0.3%) to the cultures that were adjusted at different temperatures (20-30°C). The cell cultures were allowed to continue growing for 6 hr, 8 hr and 10 hr (according to the experimental design) after induction and adjustment of growing temperature. Cells were harvested from the spent media by centrifugation (6000×*g*) at 4°C, for 20 min and stored at -20°C for further use (Ismail and Amid, 2008a; Ismail and Amid, 2008b). All experiments were performed in triplicates.

### Enzyme recovery and purification

The harvested cells were subjected to sonication (sonicator, 150 v/t model, Biologics, Inc. USA) on ice using 6-10 sec burst, with 10 sec interval at high amplitude. This was followed by centrifugation (13000×*g*) at 4°C, for 30 min and the supernatant was collected and purified by AKTA purifier FPLC system (GE Healthcare Bio-Sciences, USA). Purification of recombinant bromelain was conducted in accordance to the manufacturer's instructions. A glass column for chromatography (4.6 mm × 100 mm, Life Technologies, California) was filled with 1 mL of Ni-NTA His•Bind resin (Novagen, Germany). The FPLC system was set at flow rate of 1 mL/min. Washing step was achieved by using wash buffer (50 mM NaH_2_PO_4_, 300 mM NaCl, 20 mM imidazole, pH 8) while purified recombinant bromelain was eluted using elution buffer (50 mM NaH_2_PO_4_, 300 mM NaCl, 500 mM imidazole, pH 8).

### SDS-PAGE

After each step of enzyme recovery and purification, the protein fractions were tested by SDS-PAGE in 12.5% polyacrylamide gels as described earlier (Amid *et al*. 2011). Visualization was conducted by staining with Coomassie Brilliant blue (Laemmli, 1970).

### Determination of enzyme activity

The reaction mixture contained 0.1 mL of purified recombinant bromelain and 1.1 mL of 1% casein in 0.1 M Tris-HCl buffer (pH 8.0) with 20 mM cysteine (final concentration). The reaction was conducted at 37°C for 20 min. The reaction was stopped by adding 1.8 mL of 5% (w/v) trichloroacetic acid (TCA). This was followed by centrifugation at 10000×*g *for 15 min and the absorbance of the supernatant was measured at 280 nm. One unit (U) of the enzyme was defined as the amount of protease that produces an increment of one absorbance unit per minute in the assay conditions (Bruno *et al*. 2003).

## Results

### Screening of culture growth induction conditions using one-factor-at-a-time (OFAT) method

In order to explore the possibility of improving the expression level of recombinant bromelain, several one-factor experiments were employed to investigate the effects of various cultivation conditions on recombinant bromelain production. The tested variables included cell concentration, post-induction temperature, inducer concentration and post-induction period for cell harvest. All the experiments were carried out in triplicates.

#### Cell concentration (induction time)

**Figure 1:**
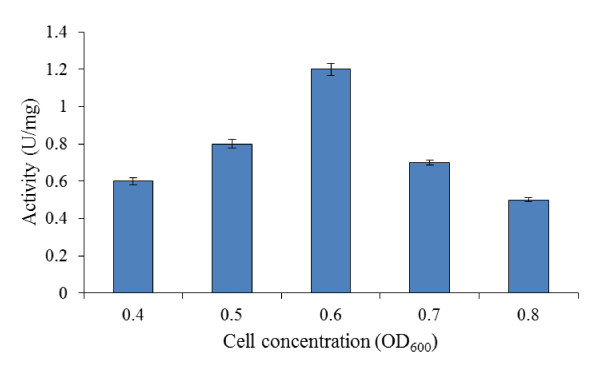
**Effects of different cell concentrations on bromelain activity by BL21-AI**. Tests were carried out at varying cell concentrations (OD_600 nm _of 0.4-0.8), while temperature of 37°C, 0.2% (w/v) L-arabinose and 4 hr post-induction period were kept constant.

Induction serves as a turning point between cell growth and recombinant protein synthesis in *E. coli *(Xu *et al*. 2006). The cloning and expression system employed requires the addition of L-arabinose to trigger the transcription of foreign gene in the plasmid and initiate the translation of recombinant protein. This leads to several changes in the metabolism of host cells. The activity of recombinant bromelain at different induction time varies widely from 0.6-1.2 U/mg (Figure 1). The maximum yield of the protein was obtained when the induction was carried out at approximate OD_600 nm _of 0.6. It can be seen from Figure 1 that early induction has great effects on the bromelain production.

#### Post-induction temperature

**Figure 2:**
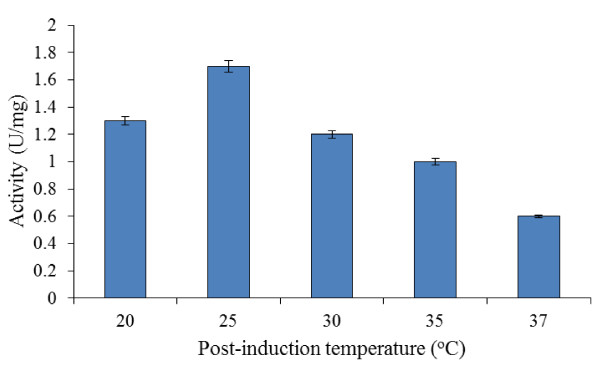
**Effects of different post-induction temperatures on bromelain activity by BL21-AI**. Experiments were conducted at various post-induction temperatures (20-37°C), with OD_600 nm _of 0.4, 0.2% (w/v) L-arabinose and 4 hr post-induction period being constant.

The effects of post-induction temperature on recombinant bromelain production are shown in Figure 2. The results obtained indicated that the target protein achieved highest activity value of 1.70 ± 0.03 U/mg at a temperature of 25°C. The bromelain activity increased as the post-induction temperature was raised from 20 to 25°C. However, the activity of the protein declined as the post-induction temperature was further raised from 30°C to the optimal growth temperature of 37°C.

#### Effects of L-arabinose concentration

**Figure 3:**
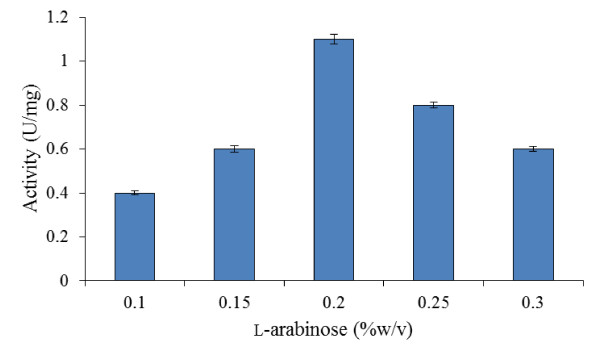
**Effects of different inducer concentrations on bromelain activity by BL21-AI**. Tests were carried out at varying L-arabinose concentrations (0.1-0.3% w/v) while OD_600 nm _of 0.4, 37°C and 4 hr post-induction period were kept constant.

As the expression vector used in this study had T7 *ara*BAD promoter, the final L- arabinose concentration should be optimized. This is necessary since *ara*BAD is a highly regulated promoter in which its expression level is essentially modulated by L-arabinose concentration (Guzman *et al*. 1995). It can be inferred from Figure 3 that the concentration of the inducer had a strong effect on the specific activity of recombinant bromelain. An increase in recombinant bromelain production was seen by increasing the L-arabinose concentrations from 0.1-0.2% (w/v). The maximum recombinant protein production was achieved at inducer concentration of 0.2% (w/v). The specific activity of bromelain decreased afterwards as higher levels of L-arabinose retarded the rate of cells' growth.

#### Post-induction period for cell harvest

**Figure 4:**
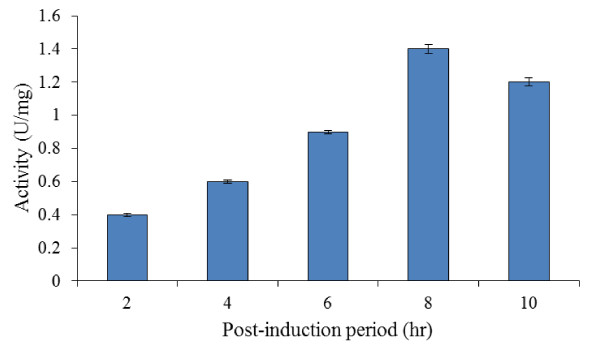
**Effects of different post-induction period (2-10 hr) on bromelain activity by BL21-AI**. Experiments were conducted at OD_600 nm _of 0.4, 37°C and 0.2% (w/v) L-arabinose.

The recombinant protein synthesis began extensively after the addition of L-arabinose into the growth culture. The production of target bromelain was found to be proportional to the expression time up to 8 hr (Figure 4). The maximum bromelain activity of 1.40 U/mg ± 0.03 was attained after 8 hr post-induction period. After 8 hr, the production rate of recombinant bromelain started to regress. It could be observed that the activity of recombinant bromelain after L-arabinose induction changed with expression conditions, especially the post-induction period.

### Optimization by Response Surface Methodology (RSM)

The use of statistically based experimental design is a vital tool in optimizing the induction conditions that may bring several fold increment in bromelain production. RSM provides several important advantages such as the factor effects, determination of the optimum values as well as development of a system model with less experimental requirements (Salihu *et al*. 2011). It could be seen from the results in Table 2 that the highest amount of bromelain produced (8.9-9.2 U/mg) was obtained in the runs representing the center points (runs 1, 6, 9, 11, 14 and 17) while the lowest value was observed in run 3 (4.8 ± 0.02 U/mg). A second order regression equation showed the dependence of bromelain activity on the growth culture induction conditions. Multiple regression analysis of the experimental data provided the parameters of the equation. A second-order polynomial equation was used to express the empirical relationship between the response and the significant variables:

(2) $$\begin{array}{c} \text{Y}\left(\text{bromelain activity},\text{ U}/\text{mg}\right)=+9.05+0.35\text{A}-0.39\text{B}+0.04\text{C}\\ -0.85{\text{A}}^{2}-0.75{\text{B}}^{2}-0.50{\text{C}}^{2}-0.84\text{AB}-0.64\text{AC}-0.56\text{BC} \end{array}$$

where the response (Y) is the bromelain production, while A, B and C are the temperature, inducer concentration and post-induction period, respectively.

The analysis of variance (ANOVA) tested using Fisher's statistical analysis, was used to verify the adequacy of the model. The results are shown in Table 3. The model F-value of 99.32 with *p*-value of 0.0001 implied that the model was significant. As lack of fit is not significant, it clearly implies that the obtained experimental responses adequately fit with the model. The predicted *R*^2 ^value of 0.9266 is found to be in reasonable agreement with the adjusted *R*^2 ^value of 0.9790 and thus, suggesting that the model is well fit. In addition, the adequate precision value of 31.695 for recombinant bromelain production implies that the model can be used to navigate the design space. In this model, a lower value of 2.32 for the coefficient of variation (CV), suggested a good precision and reliability of the experiment. The coefficient values of the regression equation are shown in Table 3.

**Table 3:** Analysis of Variance of quadratic model for bromelain production

Source	Sum of squares	F-value	P-value
Model	30.67	99.32	0.0001
Post-induction temperature, A	1.22	35.71	0.0001
Inducer concentration, B	1.52	44.34	0.0001
Post-induction period, C	0.016	0.47	0.5102
A^2^	2.01	58.54	0.0001
B^2^	1.57	45.64	0.0001
C^2^	0.70	20.41	0.0001
AB	5.61	163.56	0.0001
AC	3.25	94.77	0.0001
BC	2.53	73.78	0.0001
Lack of fit	0.21	1.54	0.3233

R^2 ^= 0.9889, adjusted R^2 ^= 0.9790, C.V. = 2.32, adequate precision = 31.695

**Figure 5:**
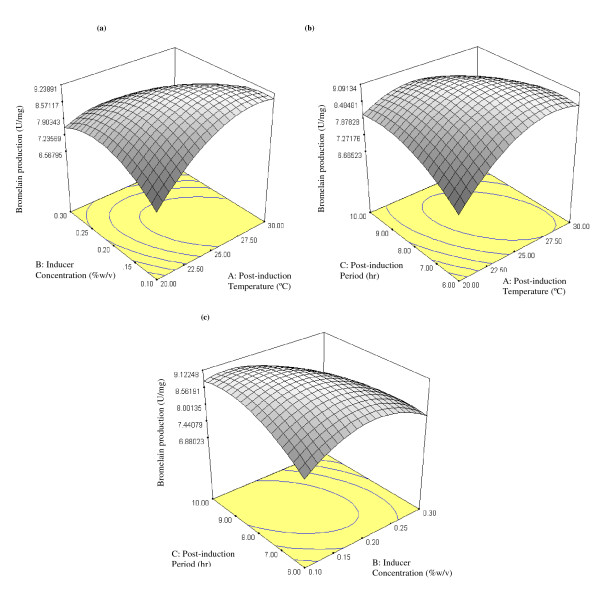
**The 3D response surface curves of the combined effects of post-induction temperature, inducer concentration and post-induction period on bromelain production**. (a) Temperature and inducer concentration at a fixed level of post-induction period. Tests were carried out at OD_600 nm _of 0.6 with varying temperature (20-30°C) and L-arabinose concentrations (0.1-0.3% w/v). Post-induction period of 8 hr was used. (b) Post-induction temperature and post-induction period at fixed level of inducer concentration. Tests were carried out at OD_600 nm _of 0.6 with varying post-induction temperature (20-30°C) and post-induction period (2-10 hr). Inducer concentration of 0.2% (w/v) was used (c) Inducer concentration and post-induction period at fixed level of post-induction temperature. Tests were carried out at OD_600 nm _of 0.6 with varying L-arabinose concentrations (0.1-0.3% w/v) and post-induction period (2-10 hr). A post-induction temperature of 25°C was used.

In order to understand the interactions of induction conditions and to find the optimum conditions required for maximum bromelain production, the 3-D response surface curves were plotted. Response surface contour plots and three dimensional graphs provide clear information on the relationship between the response and experimental levels of each variable. They also explain the type of interaction existing between the test variables and help to obtain the optimum conditions (Haaland, 1989). Three response surfaces (Figure 5) had been shown by considering all the possible combinations. The 3D plots shown in Figure 5 are based on the function of induction conditions of the two variables with the other one being kept at its optimum level. The elliptical nature of the contour plot indicates the significance of the interactions between the corresponding variables. Figure 5a shows the interaction between post induction temperature and inducer concentration. It can be seen that maximum bromelain production was attained at induction temperature of 25°C and L-arabinose concentration of 0.2% (w/v).

Moreover, Figure 5b represents the 3D plot corresponding to temperature and post-induction period. The plot exhibited an elliptical contour that suggests both optimum operating conditions and the interaction effects between the two factors are significant. Figure 5b shows that maximum bromelain production was attained at 8 hr post-induction period.

Similarly, in the case of inducer concentration and post induction time (Figure 5c); the response plot was elliptical signifying interaction between them with optimum production of the enzyme. Consequently, the optimized cultivation conditions synergistically favor higher production of bromelain.

In order to ascertain the feasibility of the application of the optimal conditions earlier generated, a validation experiment was conducted as presented in Table 4. It could be seen that some of the experimental values are slightly higher than the predicted ones. This could possibly be explained based on the experimental conditions used in the border zone of the technological space considered in FCCCD. The validation of predicted bromelain production yielded 9.6 ± 0.02 U/mg of the enzyme under optimized cultivation conditions. Hence, 5.65-fold raise in overall bromelain production was achieved as compared to non-optimized conditions (Figure 2, in which maximum bromelain activity was 1.7 ± 0.03 U/mg).

**Table 4:** Experimental model validation

Experiment	Post-induction temperature (°C)	Inducer concentration (% w/v)	Post induction period (hr)	Bromelain production(U/mg)	
				
				Experimental	Predicted
1	27	0.15	8	9.60 ± 0.02	9.20
2	27	0.15	9	9.30 ± 0.03	9.10
3	27	0.20	9	8.90 ± 0.03	8.80
4	27	0.18	9	9.10 ± 0.02	9.00
5	27	0.15	9.5	9.20 ± 0.03	9.00
6	25	0.15	9	9.30 ± 0.03	9.10

## Discussion

There are several strategies that are being employed to improve culture cultivation conditions for expressing soluble recombinant proteins in *E. coli*. The screening of experimental conditions for the improvement of enzyme expression coupled with the RSM experimental design serve as vital tool for analyzing the influence of cultivation conditions on the expression of recombinant bromelain. The design had proved to be effective in determining the important induction conditions that have significant effect on recombinant bromelain production in *E. coli *BL21-AI. Using face centered central composite design (FCCCD), the optimum induction conditions for high bromelain activity were induction temperature (27°C), L-arabinose concentration (0.15% w/v) and post-induction period of 8 hr. The results presented in this research for the analysis of cultivation conditions for the expression of recombinant bromelain corroborate the applicability of experimental design to the field of molecular biology.

This study had found out that the studied parameters had exerted great effects on the production of recombinant bromelain at higher level. Lower cultivation temperature of 25°C under optimized conditions (Figure 5a and 5b), had effectively improved the bromelain production. It is an established fact that expression of soluble recombinant enzymes is highly favored by lower growth temperature. During expression of recombinant proteins that have tendency to form insoluble aggregates in *E. coli*, decreasing the post-induction temperature has been shown to significantly reduce protein aggregation (MacDonald *et al*. 2003). In addition, in T7 expression system (used in this study), a large number of recombinant proteins often precipitate when expressed at 37°C, but tend to be soluble when induction temperature is lowered to 15-25°C (Vera *et al*. 2007). This is because slower rates of protein production allow the newly transcribed recombinant proteins sufficient time to fold properly. Hence, lower temperatures during induction in such expression system should be used as the default (Vera *et al*. 2007). This is also supported by the findings of Schein and Noteborn (1988) in which the productions of soluble fraction of the recombinant proteins expressed in various *E. coli *strains were increased at culture temperatures in the range of 20-30°C.

Moreover, temperature also affects the stability of plasmid in recombinant *E. coli *cultures (Shin *et al*. 1997 &Wang *et al*. 2005) and thus affects production of soluble proteins (Islam *et al*. 2007 &Swalley *et al*. 2006). Lower temperatures coupled with lower cell growth rates usually favor higher production of soluble protein. This is based on the type of expression system and recombinant protein involved (Urban *et al*. 2003). The results from the aforementioned study (Urban *et al*. 2003) are in well accordance with our findings.

In this study, it was discovered that moderate concentration of L-arabinose (0.2% w/v) used under the optimized conditions, has greatly contributed towards attaining higher bromelain production as shown in Figure 3, 5a and 5c. This indicated that higher levels of L-arabinose decreases recombinant protein expression. In fact it has been reported that higher concentrations of L-arabinose cause over production of recombinant protein that leads to ribosomal destruction, production of heat shock proteins and eventually cell death (Guzman *et al*. 1995). Our findings are consistent with the works of Manderson *et al*. (2006) in which maximum recombinant aspin production was attained at L-arabinose concentration of 0.2%(w/v) but reduced at higher inducer concentration. Aspin is an aspartyl protease inhibitor homolog which is being produced by the parasitic nematode, *Trichostrongylus colubriformis *(Manderson *et al*. 2006). In addition, the use of partial induction to slow the rate of recombinant protein expression has been reported to enhance the formation of soluble protein (Lim *et al*. 2000). More so, a significant increase was observed in the production of soluble interferon upon induction with low level of L-arabinose (Lim *et al*. 2000).

The optimization studies in this work were conducted at a fixed cell concentration (induction time) which was screened earlier under OFAT method. Maximum bromelain production was achieved at cell concentration of OD_600 nm _= 0.6 as shown in Figure 1. The effects of induction time depend on the specific expression case (Manderson *et al*. 2006). In some cases, early induction increases soluble protein production of some recombinant proteins by limiting the culture growth rate (Delisa *et al*. 2001). This was supported by the findings of Lim and Jung (1998) in which a five-fold improvement in soluble interferon production was attained by induction in early logarithmic phase. However, Akesson *et al*. (2001) had reported higher levels of recombinant protein expression when induced in the late exponential growth phase.

The effect of post-induction period on bromelain production was found to be at its peak after 8 hr as shown in Figure 4, 5b and 5c. Bromelain production started to reduce beyond this period. Thus, cultivation at lower temperatures and moderately longer post-induction period could significantly enhance the expression of the recombinant protein in comparison with the standard *E. coli *growth temperature of 37°C. This could be explained by the fact that the solubility and refolded conformation of recombinant enzymes were greatly enhanced by cultivation conditions and certain post-translational time was needed to get a mature protein with high enzymatic activity (Xu *et al*. 2007). It is vital to control the time and extent of plasmid gene expression as the recombinant protein can effectively inhibit host cell growth, presumably due to its toxicity (Shin *et al*. 1997). Moreover, the metabolic load exerted on the host cells through heterologous gene expression may lead to retardation of growth (Manderson *et al*. 2006).

## Competing interests

The authors declare that they have no competing interests.

## End notes

1. All the tests were carried out at 37°C, OD_600 nm _of 0.4, 0.2% (w/v) L-arabinose and 4 hr post-induction period (besides the specific tested conditions).

2. Cultivation conditions and their ranges were selected based on literature reports on recombinant proteins expression.
